# Persistent self-reported health complaints in Norwegians who attribute their symptoms to tick bites or tick-borne disease– a cross-sectional controlled study

**DOI:** 10.1186/s12879-025-11104-0

**Published:** 2025-05-16

**Authors:** Audun Olav Dahlberg, Audun Aase, Harald Reiso, Erik Thortveit, Randi Eikeland, Morten Engstrøm, Rune Midgard

**Affiliations:** 1 Norwegian University of Science and Technology, Trondheim, NO-7491 Norway; 2 Department of Neurology and Clinical Neurophysiology, St. Olav Hospital Trust, Trondheim, NO-7006 Norway; 3 Department of Neurology, Molde Hospital, Møre and Romsdal Hospital Trust, Parkvegen 84, Molde, NO-6412 Norway; 4 Department of Method Development and Analytics, Norwegian Institute of Public Health, Oslo, NO-0213 Norway; 5 Norwegian National Advisory Unit on Tick-borne Diseases, Sørlandet Hospital Trust, Post-box 783, Arendal, NO-4809 Norway; 6 Department of Neurology, Sørlandet Hospital Trust, Post-box 416, Kristiansand, NO-4604 Norway; 7 Institute of Health and Nursing Science, University of Agder, Post-box 422, Kristiansand, NO-4604 Norway

**Keywords:** Persistent health complaints, Lyme borreliosis, Serology, PROM, Cross-sectional controlled study

## Abstract

**Background:**

The frequency and mechanisms of persistent health complaints attributed to tick bites or tick-borne diseases are unknown. We evaluate such complaints in Norwegian cases and controls.

**Methods:**

People older than 18 years with persistent health complaints of six months or more attributed to tick bites or tick-borne diseases (cases) were recruited into a nationwide cross-sectional study between October 2016 and January 2021. Demographic data, tick bites, antibiotic use, and tick-borne pathogen serology were recorded. We evaluated somatic symptoms (PHQ-15), fatigue (Fatigue Severity Scale), mental and physical health (RAND-36), affective symptoms (HAD Scale) and modern health worries (MHW Scale) as outcome measures. Serological tests included IgG antibodies against *B. burgdorferi (Bb)* and other tick-borne pathogens. The control population (*n* = 2803) was recruited from a tick-endemic region in Søgne, southern Norway. Differences between cases and controls were evaluated.

**Results:**

A total of 500 responses were collected through general practitioners (*n* = 14), by invitation (*n* = 94), and by Short Message Service (SMS) (*n* = 392). The estimate of prevalence is based on 392 of 270.000 included by SMS (0.15%). The SMS cohort reported better physical health than those recruited by invitation. Cases had significantly more somatic and affective symptoms, fatigue, comorbidities, and reduced quality of life related to health than controls. The differences in fatigue and physical health between cases and controls were not related to previous tick exposures. *Bb* IgG and other antibodies against tick-borne pathogens were more prevalent in cases than controls. In multivariable analyses, cases that were never treated did not exhibit higher somatic symptom scores compared to those treated multiple times. Seropositive *Bb* cases had worse mental health (*p* < 0.001) and more depressive symptoms (*p* = 0.017) than seronegative cases.

**Conclusions:**

The crude prevalence of persistent health complaints in Norway attributed to tick bites or tick-borne diseases is 0.15%. The cases reported significantly poorer physical health, including increased fatigue, when compared to the controls. These relationships were not affected by tick exposures. However, poorer mental health in cases may be associated with *Bb* seropositivity, especially for the ones with comorbidities. In conclusion, no clear associations were found between tick bites, tick-borne diseases and persistent health complaints.

**Supplementary Information:**

The online version contains supplementary material available at 10.1186/s12879-025-11104-0.

## Background

European Lyme borreliosis (LB) is a tick-borne infection caused by spirochetes from the *Borrelia burgdorferi (Bb)* sensu lato complex that include *B. afzelii*,* B. garinii*, and *B. burgdorferi* sensu stricto. According to European guidelines, disseminated stages of LB are diagnosed by typical clinical manifestations and laboratory evidence of *Bb* infection [1]. In daily practice, the diagnosis of LB can be challenging. The available serological tests have limitations in distinguishing between current, long-lasting, or past infections [2]. The recommended treatment of disseminated LB is two weeks of antibiotics, in some cases longer depending on the type of disseminated LB, severity, and duration of the disease before diagnosis [1, 3]. However, because a subset of patients with LB report long-lasting health problems (fatigue, lethargy, headache, arthralgia, cognitive and musculoskeletal problems, and reduced quality of life) after standard antibiotic treatment, the restricted duration of antibiotic treatment has been challenged [3–5]. The mechanisms underlying persistent health complaints and their relationship to LB remain unclear. While most clinicians attribute these health complaints to sequelae, (post-Lyme disease syndrome - PTLDS) [1, 3], some clinicians and patients believe these symptoms may result from persistent infection [6], co-infections with other tick-borne pathogens [7, 8], abnormalities in the host immune system [9] or psychological factors [10, 11]. Persistent symptoms attributed to LB have not been evaluated in a population-based study in Norway, and the prevalence of people attributing health problems to tick bites or tick-borne diseases is unknown. Some people have received repeated and long-term treatments with antibiotics and substances that have not been adequately tested for efficacy [12, 13]. People who perceive themselves as sick from tick bites or previous tick-borne diseases may experience lack of recognition and poor specific knowledge of their symptoms in the health system [14]. Different interpretations of symptoms by patients and doctors may lead to frustration and reduced trust in the healthcare system, and lead patients to seek advice elsewhere, such as less trustworthy web pages.

We aimed to estimate the prevalence of people with persistent health complaints attributed to tick bites or tick-borne diseases in Norway (cases) and investigate associations between persistent health complaints, patient-reported outcome measures (PROM), IgG antibodies to different tick-borne pathogens, tick bites, self-reported tick-borne diseases, and antibiotic treatments.

## Materials and methods

### Inclusion criteria, blood samples, and sample size calculations

**Fig. 1:**
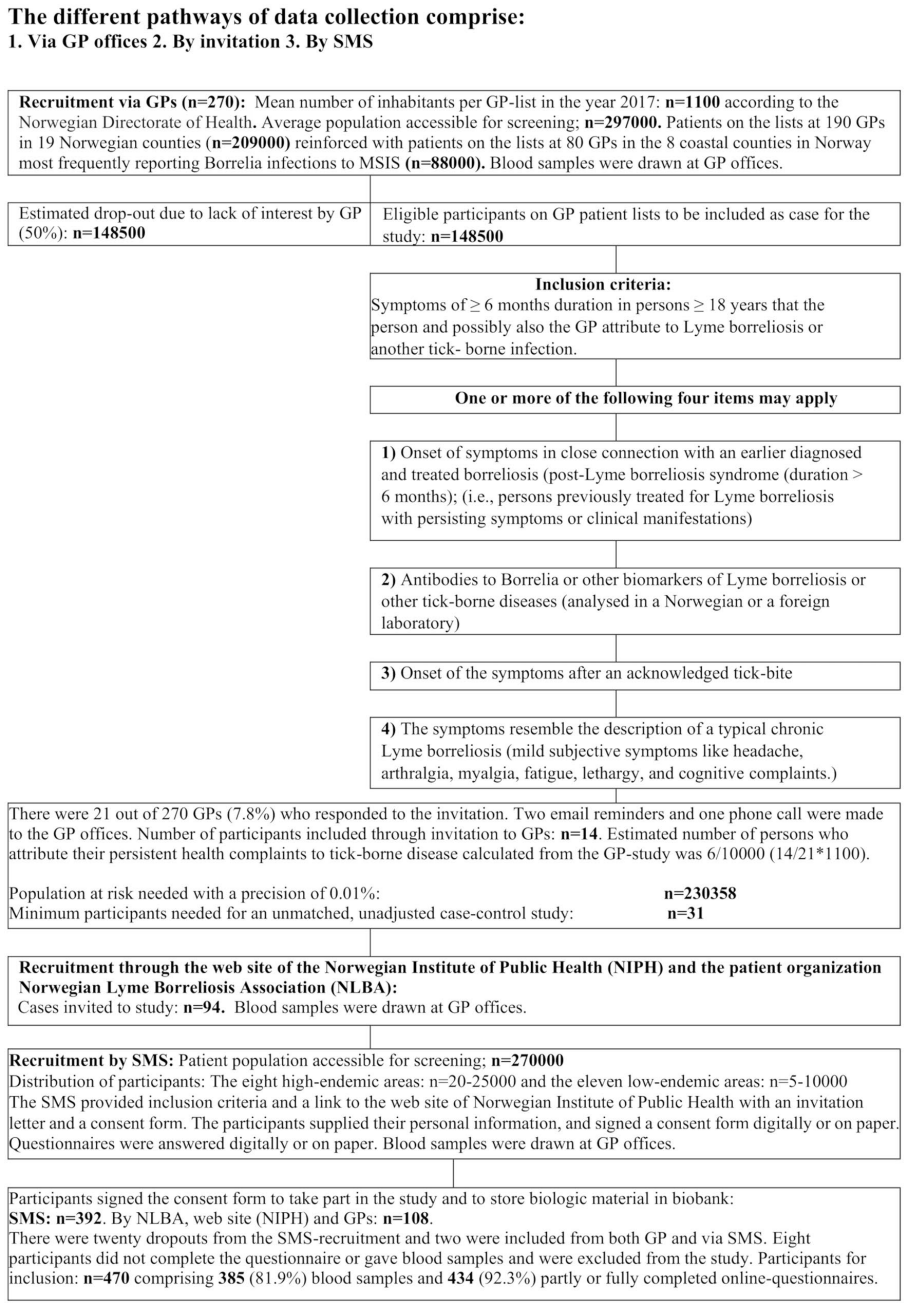
Inclusion and selection process in the study

Details on inclusion and sample size are shown in Fig. 1, which includes the terms ‘chronic Lyme disease’ and ‘post-Lyme disease syndrome’ as previously published [3, 15, 16]. Blood samples were drawn in the general practitioner (GP) office of all individual participants. The sample size estimation for this cross-sectional study [17] was based on the estimated prevalence obtained through our recruitment method via Norwegian GPs (See the sections on recruitment from GPs, response from the cases, and demographics. ). A seroprevalence of 22% for *Bb*-IgG was previously found in a cohort living in the municipality of Søgne [18]. We assumed at least 50% *Bb* seropositivity in cases with persistent symptoms, according to a randomized controlled trial in the Netherlands [19]. To detect statistically significant differences between cases and controls in an unmatched study without accounting for confounding variables, we needed 31 participants with α < 5%, β > 80% and with a control / case ratio of 4:1 [20]. The selection of participants described in the cases section  was based on these calculations.

### The cases

#### Recruitment through a selection of general practitioners (GPs)

Participants were recruited primarily through an open national invitation to 270 Norwegian GPs. The physicians were randomly selected from a list supplied by the Norwegian Health Economics Administration (HELFO) sorted by county. LB occurs primarily along the Norwegian coast as far north as the southern part of Nordland County according to the Norwegian Surveillance System for Communicable Diseases (MSIS) in 2018. Therefore, we invited 10 physicians from each of the 11 low-endemic counties and 20 physicians from each of the eight high-endemic coastal counties (ranging from Vestfold through Møre and Romsdal) to report their cases, thus strengthening the clinical material from the coastal counties. The physicians in these high-endemic regions were responsible for 72–80% of the reported patients with disseminated LB in Norway from 2010 to 2015. The recruitment was carried out between October 2016 and August 2017.

#### Recruitment through the website of the Norwegian National Advisory Unit on Tick-borne Diseases

Due to the low number of participants recruited through GP contacts, an additional recruitment strategy was chosen in cooperation with the Norwegian Lyme Borreliosis Association (NLBA), a national interest group for patients with LB. Information about our study and an invitation to participate was published in newspapers and on the websites of both the Norwegian National Advisory Unit on Tick-borne Diseases and the NLBA. Participants were recruited between August 2017 and June 2018.

#### Recruitment through the short message service (SMS)

According to Statistics Norway (year 2017), 98% of the Norwegian population owned a cell phone, 89% owned a smart phone, and 97% had access to the Internet. By random selection of people over 18 years of age from the Norwegian National Population Registry (NPRN), we invited 5,000–10,000 people from each of the eleven low-endemic counties, and 20,000–25,000 individuals from the highest endemic areas for Lyme borreliosis. The sample size of 270,000 was based on the estimated prevalence of the GP cohort (Fig. 1). A brief introduction of the National Institute of Public Health (NIPH) study by SMS with an accompanying link to the forms included the text:

Hi. Do you have persistent symptoms after tick bites? Join our study on borreliosis on the NIPH website. The main inclusion criteria are being 18 years or older and having symptoms of at least 6 months’ duration that you or your doctor believe are related to Lyme borreliosis or another tick-borne infection.

The participants received an informative text and were asked to report if they met one or more of the inclusion criteria. Participants received supplementary information and a consent form. After signing the informed consent digitally by BankID^®^ or manually by mail correspondence, participants were referred to an electronic questionnaire by SMS or email. Individuals who did not have the ability to communicate on digital platforms received a letter containing the questionnaire.

The recruitment of participants was carried out between December 2019 and January 2021.

### The controls

Participants in a study conducted in Søgne municipality, a high-endemic area of ticks and tick-borne diseases in southern Norway, served as asymptomatic controls. The Søgne study was carried out between June 2015 and June 2016. Asymptomatic controls selected from the Søgne cohort stated that they had no health complaints attributed to tick-borne disease (*N* = 2803). The cohort is described elsewhere [18, 21, 22].

### Clinical variables and questionnaire

Cases and controls completed an online or a paper version of the questionnaires. For the SMS cohort, 280/363 (77.1%) participants completed their online questionnaire, 9/363 (2.5%) partially answered, while 47/363 (12.9%) completed a paper version. Some did not respond to the questionnaires but provided a blood sample (27/363 (7.4%)). For the 107 participants recruited through the GP or by invitation, only online questionnaires were accessible. Among these, 97/107 (90.7%) completed the questionnaires and one participant partly completed the questionnaire. We recorded demographic data, physical activity (Table 1), exposure to tick bites and previous tick-borne infections, antibiotic treatment for tick-borne bacterial infections (Table 2), comorbidities and regular medications (Table 3). For details, consult the supplementary questionnaire. Cases and controls completed the Patient Health Questionnaire-15 (PHQ-15) to assess the burden of somatic symptoms [23], the Fatigue Severity Scale (FSS) [24] and the RAND-36 health-related quality of life (HRQoL) survey [25]. The results of the 36 questions of RAND-36 are combined in a physical component summary (PCS) and a mental component summary (MCS). These scores assess physical and mental health. We also recorded the Hospital Anxiety and Depression Scale (HADS) [26] and the Modern Health Worries Questionnaire (MHW) [27]. For PHQ-15, a score between 0–4 is normal, 5–9 is mild, 10–14 is moderate, and 15–30 is a burden of severe symptoms. In FSS, a scale between 1 and 7 ranges from ‘strongly disagree’ to ‘strongly agree’, and a calculated mean score > = 4 implies severe fatigue. RAND-36 has a scale of 0 to 100 where a high score indicates good health. Both HADS depression and HADS anxiety range from 0 to 21 points, and a mean score > = 8 indicates borderline symptoms. In MHW, a scale of 0–5 differentiates between no concerns and deep concerns. We also recorded previous tick-borne infections, including erythema migrans (EM) > = 5 cm in diameter, Lyme neuroborreliosis (LNB), Lyme arthritis, and other unspecified tick-borne infections. The vaccination status against tick-borne encephalitis virus (TBEV) was recorded.

**Table 1:** Demographics. Gender, age, way of recruitment, nationality, education, employment, income, and physical activity

	Cases	Controls	*p*-value
***Participants (N)***	470	2803	
***Gender***			0.935
*Male*	190 (45.2)	1274 (45.5)	
*Female*	280 (54.8)	1529 (54.5)	
***Age (mean***,*** 95%CI)***:	54.4 [53.0–55.8]	48.5 [48.0–49.0]	**< 0.001**
Recruitment*			
*GP offices*	14 (3.0)	1182 (42.3)	**< 0.001**
*By invitation*	93 (19.8)	1612 (57.7)	**< 0.001**
*Short Message Service (SMS)*	363 (77.2)	NA	
***Nationality***			
*Norwegian*	405 (96.4)	2681 (95.9)	0.578
Education after primary school**			
*< 3 years*	107 (25.7)	1045 (37.5)	**< 0.001**
*3–6 years*	174 (41.8)	1059 (38.0)	0.134
*> 6 years*	122 (29.3)	600 (21.5)	**< 0.001**
*Student*	13 (3.0)	83 (3.0)	0.870
***Employment***			
*Fully employed*	123 (29.6)	1498 (53.4)	**< 0.001**
***Net income/month: > 20.000 NOK***	381 (91.4)	2489 (89.1)	0.158
***Physical activity > 3 h per week***	243 (57.9)	1559 (55.7)	0.415

* Among the cases, *n* = 363 (77.2%) were recruited via Short Message Service (SMS) as outlined in M&M section on recruitment through SMS.

NA = not applicable. Fractions of people exposed in the column and given percent (%). ** Norwegian primary school lasts for ten years.

**Table 2:** Tick bites and erythema Migrans, disseminated borreliosis, antibiotic treatment, vaccination, comorbidities, and concomitant medication

	Cases	Controls	*p*-value
***Tick bites and erythema migrans***			
*Tick-bite more than twice*	306 (73.0)	2013 (72.0)	0.652
*Tick-bite last year*	148 (35.3)	918 (32.9)	0.322
*Erythema migrans twice or more*	69 (16.7)	188 (6.7)	**< 0.001**
***Disseminated borreliosis***			
*Neuroborreliosis*	97 (22.5)	17 (0.6)	**< 0.001**
*Borrelia arthritis*	49 (11.4)	8 (0.3)	**< 0.001**
TBE*	10 (2.3)	0 (0.0)	**< 0.001**
*Other Bb infection*	86 (19.9)	38 (1.4)	**< 0.001**
*Tick-borne disease not specified*	48 (11.2)	18 (0.6)	**< 0.001**
*One or more episodes of disseminated* *borreliosis*	220 (50.2)	76 (2.7)	**< 0.001**
*No previous borreliosis* ***Antibiotic treatment and vaccination***	73 (17.8)	2117 (75.8)	**< 0.001**
*More than two antibiotic treatments against tick-borne disease*	168 (41.7)	109(4.0)	**< 0.001**
*One antibiotic treatment*	154 (38.2)	278 (10.3)	**< 0.001**
*No antibiotic treatments*	81 (20.1)	2318 (85.7)	**< 0.001**
*Fully vaccinated against TBE*	26 (6.7)	73 (2.6)	**< 0.001**
***Comorbidities and concomitant medication***			
Comorbidities**	177 (42.1)	656 (23.4)	**< 0.001**
Concomitant medication***	148 (35.3)	701 (25.0)	**< 0.001**

* Tick-borne encephalitis virus infection

** Comorbidities are defined as two or more diseases or medical conditions and is outlined in Table 3

*** More than one concomitant medication against a medical condition

Fractions of people exposed in the column and given percent (%)

*P*-values calculated using the chi-square or Fisher’s exact test, when appropriate. NA means not applicable

**Table 3:** Comorbidities in cases and controls

	Cases	Controls	*p*-value*
*Neurological disease*	62 (14.8)	105 (3.7)	**< 0.001**
*Rheumatologic disease*	101 (24.0)	356 (12.7)	**< 0.001**
*Endocrinological disease*	43 (10.2)	233 (8.3)	0.188
*Psychiatric disease*	57 (13.6)	298 (10.6)	0.073
*Cardiovascular disease*	31 (7.4)	172 (6.1)	0.327
*ME/CFS*	70 (16.5)	32 (1.1)	**< 0.001**
*COPD / asthma*	31 (7.3)	167 (6.0)	0.279
*Cancer*	23 (5.4)	143 (5.1)	0.787
*Dermatological disease*	45 (10.5)	140 (5.0)	**< 0.001**
*Ophthalmological disease*	30 (7.1)	95 (3.4)	**< 0.001**
*Allergies*	79 (18.4)	533 (19.0)	0.751
*Other diseases*	94 (22.1)	208 (7.4)	**< 0.001**

* Fractions and given as valid percent (%) if responded. *P*-values calculated by the chi-square test

### Serological tests

We received blood samples from 385/470 (81.9%) of the included persons and 2800/2803 (99.9%) of the controls. Serum IgG antibodies against *Bb* sensu lato were measured using the enzyme-linked immunosorbent assay kit Enzygnost^®^ Lyme link VIsE/ IgG (ELISA) (Siemens Healthcare Diagnostics Products GmbH, Erlangen, Germany) (subjects recruited by general practitioners and by invitation). This test was no longer available (discontinued by the manufacturer) when we received sera from the SMS-study; therefore we switched to the Serion ELISA classic *Bb* IgG (subjects from SMS recruitment). Parallel examination of serum panel between these two kits revealed excellent agreement. The cut off limit for IgG antibodies for *Bb* was set to > 5 U/ml. Samples with an equivocal score were classified as negative. IgG antibodies against TBEV, *F. tularensis*, and *C. burnetii* phase 2 antigen were analysed with SERION ELISA classic kits (Serion Diagnostics, Institut Virion/ Serion GmbH, Wurzburg, Germany) according to the manufacturer’s instructions. The classification of sera as negative, equivocal, and positive was performed according to the kit instructions. Indirect immunofluorescent assay (IFA) tests were used for the detection of serum IgG antibodies against *Anaplasma phagocytophilum* (*Anaplasma phagocytophilum* IFA IgG), *Babesia microti* (*Babesia microti* IFA IgG), *Bartonella henselae* and *quintana* (*Bartonella* IFA IgG) from Focus Diagnostics of Cypress, California, USA, and *Rickettsia helvetica* and *conorii* (*Rickettsia* Screen IFA IgG Antibody Kit) and *Babesia divergens* (*Babesia divergens* IgG IFA Kit) from Fuller Laboratories, Fullerton, California, USA. Due to substantial cross-reactivity for IgG antibodies within *Bartonella henselae/quintana* and within the *Rickettsia helvetica/conorii*, the results are summarised for the two *Bartonella* species and the two *Rickettsia* species. Analyses and interpretation of results were performed according to the manufacturer’s instructions. A screening dilution of 1:64 was applied for the evaluation of IFA tests. The IFA slides were evaluated separately by two investigators (only one investigator for the SMS cohort). Positive sera were titrated further to give an end titre. Bartonella and Coxiella were included due to public awareness that these microbes could be potential tick-borne agents, despite no established link to tick bites.

### Statistical analyses

For unadjusted two-group comparisons between independent variables, we used the chi-square test or Fisher’s exact test for categorical variables and independent Student’s t test, Mann-Whitney U test or ANOVA statistics for continuous variables. We used binary logistic regression and multiple linear regression with adjustment of potential confounders, i.e. age, sex, education, physical activity, and comorbidities. The clinical variables PHQ-15, FSS, RAND-36 and HAD scores were defined as separate outcome measures. Binary logistic regression was performed to assess odds ratios (OR) between cases and controls (group variable) for different exposures to ticks and outcome measures adjusted for confounders.

The outcome measures were defined as dependent variables in the multiple linear regression models and the estimated marginal means (EMM) with 95% confidence intervals were calculated. Differences between recruitment methods on clinical outcome variables were evaluated with interactions between the group and recruitment methods. We performed multiple linear regressions to assess whether the outcome variables differed between groups based on tick bites, antibodies to tick-borne pathogens (serology), self-reported tick-borne diseases, and antibiotic therapy for tick-borne infections (interaction term). Due to missing values up to 20.4% for the PHQ-15 questionnaire, multiple imputation of missing data was performed using the fully conditional specification with the Markov chain Monte Carlo method and predictive mean matching [28, 29]. Twenty-five data sets were imputed separately for cases and controls and then merged. Estimates (EMM and 95% confidence intervals) were combined using Rubin‘s rules [30]. Littles’ test assessed whether the data were missing completely at random (MCAR). Multiple comparisons were not adjusted for, as they can reduce false positives but increases false negatives [31]. The outcome variables of the control group were compared with normative data [32–35]. Statistical analyses were performed using IBM SPSS for Windows (version 29.0) and STATA (version 18.0). Microsoft Excel was utilized to generate figures and tables for the estimates from the multiple imputation model. A level of significance was established at α < 5%.

## Results

### Response from the cases

A total of 500 responses were collected. Two cases were included by SMS in addition to invitation (duplicates). Therefore, 391 cases were included by SMS and 107 cases through their GPs (14) and by invitation (93). Nineteen participants recruited by SMS withdrew from the study and one died. Eight did not complete questionnaires or provide blood samples. This led to 470 cases being eligible for the study. We obtained complete or partially completed questionnaires from 434 cases (92.3%) and blood samples from 385 cases (81.9%). See Fig. 1 for further details.

### Demographics, clinical manifestations, and comparison with normative data

The crude prevalence of persistent health complaints attributed to tick bites or tick-borne diseases was 0.06% (14/23.100) in Norwegian GP offices and 0.15% (392/270.000) in the general population. The cases (mean 54.4 years) were significantly older than the controls (mean 48.5) (*p* < 0.001). The gender distribution did not differ between cases and controls. In adjusted analyses, the differences between cases and controls in outcome measures did not change whether they were recruited by invitation or by GPs or not. However, SMS-recruited cases had better physical health (PCS) compared to invitation-recruited cases (*p* < 0.001). There were no differences in age distribution and comorbidities between the three different recruitment methods in the cases. Controls had lower MCS, and slightly higher HADS-anxiety compared to normative data; others were normal (Table S1). The demographic profile of the study population, including the recruitment method, is presented in Table 1. The clinical data for cases and controls including antibiotic treatment for tick-borne infections, vaccination, comorbidities, and concomitant medications are summarized in Table 2. Comorbidities are described in Table 3. Unadjusted differences in outcome between cases and controls are shown in Table 4.

**Table 4:** Health-related questionnaires, health-related quality of life and modern health worries in cases and controls

	Cases	Controls	*p*-value
PHQ-15* >= 10	219 (58.6)	414 (14.8)	**< 0.001**
*FSS > = 4*	305 (81.3)	770 (33.5)	**< 0.001**
*HADS depression > = 8*	109 (26.5)	257 (9.3)	**< 0.001**
*HADS anxiety > = 8*	124 (30.1)	490 (17.8)	**< 0.001**
RAND-36**			
*Mean physical component summary (PCS)*	38.9 95% CI [38.1–39.8]	48.8 95% CI [48.4–49.1]	**< 0.001**
*Summary of the mean mental components (MCS).*	45.2 95% CI [44.5–46.1]	51.05 95% CI [50.7–51.4]	**< 0.001**
*Modern Health Worries (MHW)*			
*Mean MHW*	2.00 95% CI [1.93–2.08]	1.99 95% CI [1.96–2.02]	0.597

* PHQ-15– Patient Health Questionnaire; FSS - Fatigue Severity Scale; HADS - Hospital Anxiety and Depression Scale

**RAND-36– RAND-36 Item Short Form Health Survey

Continuous variables are presented as mean with 95% confidence intervals and group variables as numbers and percent (%). Statistical analyses with chi-square, Student’s t test and Fisher’s exact test and its *p*-values

### Serological analyses

The results of the serological analyses in cases and controls are shown in Table 5.

**Table 5:** Serological analyses in cases and controls *

Serological Analyses	Cases	Controls	*p*-value
*Borrelia burgdorferi*	175/385 (45.5)	626/2800 (22.4)	**< 0.001**
*Anaplasma phagocytophilum*	13/385 (3.4)	114/1088 (10.5)	**< 0.001**
*Bartonella*	3/385 (0.8)	2/1089 (0.2)	0.115
*Rickettsia*	42/385 (10.9)	41/1085 (3.8)	**< 0.001**
*Coxiella burnetii*	0/385 (0.0)	1/73 (1.4)	0.159
*Tick-borne encephalitis virus*	35/385 (9.1)	35/1092 (3.2)	**< 0.001**
*Fransiscella tularensis*	22/385 (5.7)	3/73 (4.1)	0.781
*Babesia divergens*	14/285 (4.9)	NA	NA
*Babesia microti*	9/385 (2.3)	26/1152 (2.3)	0.927

*P-values calculated by Chi-squared or Fisher’s exact test when appropriate

NA means not applicable. The numbers in parentheses are percent (%)

There was a significant difference in the proportion (*p* < 0.001) of antibodies against more than one tick-borne pathogen between cases (32.1%) and controls (7.5%). Furthermore, 17.5% of the cases had IgG antibodies against *B. burgdorferi* sensu lato in combination with antibodies against one or more tick-borne pathogens. In controls, 2.1% had a similar combined antibody pattern (*p* < 0.001). Complete vaccination against TBEV was found in 26 of 389 cases (6.7%) and in 73 of 2797 controls (2.6%). Among the TBEV seropositive, 27 of 32 (84.4%) cases and 19 of 35 (54.3%) controls were partially or fully vaccinated.

### A comparison between cases and controls using adjusted odds ratios– associations with PROMs, health worries, tick exposures, and antibiotic treatments

Cases had significantly higher risk (*p* < 0.001) of having more than moderate somatic symptoms (8.0 [5.6–11.3]), severe fatigue (8.7 [5.9–12.8]), reduced physical (11.4 [7.4–17.6]) and mental health (3.6 [2.7–4.8]), and borderline depression (3.3 [2.3–4.7]) and anxiety symptoms (1.6 [1.2–2.3], *p* = 0.003). Modern health worries score (MHW) was not associated with an increased risk of being a case. The cases had a higher risk of positive *Bb* IgG antibodies (2.2 [1.6–3.0]), one or more tick-borne pathogens, excluding *Bb* (4.5 [3.0–6.7]), and combined *Bb* with other tick-borne pathogens (12.9 [8.2–20.2]) (all *p* < 0.001). The cases were also at increased risk of having a history of disseminated borreliosis (LNB, Lyme arthritis), TBE, or other unspecified borrelia diseases (38.1 [27.6–2.6], *p* < 0.001). The number of episodes of EM was strongly associated with being a case (*p* < 0.001); those with one episode had the highest risk (4.6 [3.5–5.9]), while those with two episodes had a lower risk (3.7 [2.6–5.3]). There was a higher risk of being a case with a known tick bite (10.7 [5.5–21.0], while there was a lower risk of being a case with two or more bites (4.8 [2.5–9.2]) (both *p* < 0.001). There was a lower risk of being a case if treated once with antibiotics for a tick-borne disease (16.1 [11.5–22.5]), compared to those treated more than twice (43.0 [30.0–62.0]) (both *p* < 0.001). Finally, there was a higher risk of being a case for those with two or more comorbidities (3.1 [2.2–4.4] (*p* < 0.001)). The multiple-imputation model yielded similar results.

### Associations between tick bites, IgG levels, and previous antibiotic therapy for tick-borne diseases with PROMs: multivariable analyses between cases and controls

**Fig. 2:**
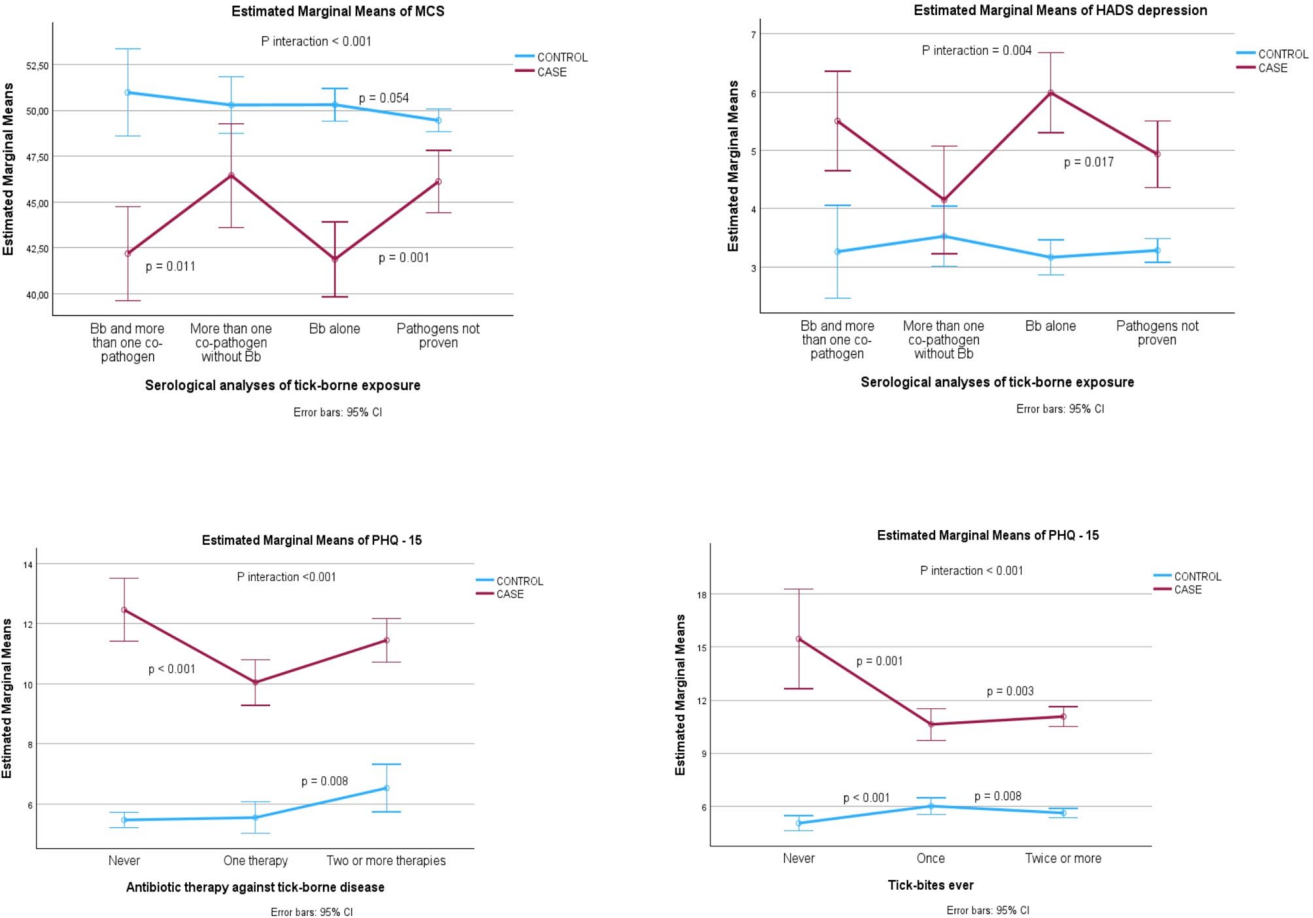
The interaction between group (cases and controls), serological results, antibiotic therapy, and tick bites Upper left: Multiple linear regression on complete cases analyses of mental component summary (MCS) on group * tick-borne pathogens Upper right: Multiple linear regression on complete cases analyses of depressive symptoms (HAD) on group * tick-borne pathogens Lower left: Multiple linear regression on complete case analyses of PHQ-15 on group * antibiotic therapy Lower right: Multiple linear regression on complete case analyses of PHQ-15 on group * tick bites P-values are shown with ‘Never’ and ‘Pathogens not proven’ (IgG negative) as the reference categories

Somatic symptoms in cases and controls were influenced by the number of antibiotic treatments (*p* < 0.001). The burden of somatic symptoms did not change significantly between cases with two or more antibiotic treatments against tick-borne infections compared to cases never treated. However, there was a significantly higher burden of somatic symptoms in cases never treated (12.5 [11.4–13.5]) compared to cases treated once (10.0 [9.3–10.8]) (*p* < 0.001). In controls, those with multiple treatments against tick-borne infections had more symptoms (6.5 [5.7–7.3]) than those who had never been treated (5.5 [5.2–5.7]) (*p* = 0.008). However, the multiple imputation model indicated a slightly higher burden of somatic symptoms in untreated cases compared to those who received two or more antibiotic treatments. The burden of somatic symptoms in cases and controls differed by the number of tick-bites (*p* < 0.001), with more than two tick bites reported a lower burden of symptoms compared to those without tick bites (*p* = 0.003). Furthermore, the multiple imputation model could not verify these differences. The differences between cases and controls in mental health (*p* < 0.001) and depressive symptoms (*p* = 0.04) varied according to the serological results. In these cases, we found worse mental health scores (*p* = 0.001) in seropositive *Bb*-IgG (41.9 [39.9–43.9]) compared to *Bb*-IgG negative (46.1 [44.4–47.8]). Significantly more depressive symptoms (*p* = 0.017) were observed in cases with *Bb* antibodies (6.0 [5.3–6.7]) compared to cases negative for IgG (4.9 [4.4–5.5]). The multiple-imputation model showed the same tendencies. The differences between cases and controls on the other outcome measures did not vary by the number of tick bites, the presence of antibodies to *Bb* or other tick-borne pathogens, self-reported tick-borne diseases, or antibiotic treatments against tick-borne disease. See Fig. 2, and Tables S2-S4 and Figures S1-S4 for details.

### Subgroup analyses of selected case groups associated with previous antibiotic treatment

Given a higher severity of symptoms in cases never treated for tick-borne infection (Fig. 2 and Figure S1), we classified the variable ‘antibiotic treatment’ into two groups: no antibiotic treatments (untreated) and one or more antibiotic treatments (treated) for tick-borne bacterial infections. In the univariate analysis, the proportion of cases with more than moderate somatic symptoms was higher in untreated cases (72.2%) than in treated cases (55.4%), with a *p-*value of 0.01. We did not find significant differences between the two treatment groups with respect to tick bites, the level of IgG antibodies to *Bb* alone or other tick-borne pathogens alone. A higher proportion of the combination of antibodies to *Bb* and other tick-borne pathogens was found among treated (*p* = 0.006) versus untreated cases. The treated group had significantly higher proportions (*p* < 0.001) of EM (65.9%) than the untreated group (43%). Among the treated cases, 15.6% reported LNB diagnosed after lumbar puncture, and among the untreated, one person reported previous LNB. Furthermore, the treated group of cases reported significantly (*p* = 0.025) more Lyme arthritis (14.8%) than the untreated group (5.2%). There was a proportion of 71.2% with one or more comorbidities among treated and 82.9% among untreated cases (*p* = 0.04). The untreated were younger than the treated (*p* = 0.029). Among the untreated cases, we found no significantly higher burden of somatic symptoms among those exposed to tick bites, tick-borne diseases, and comorbidities compared to those not exposed. See Tables S5-S6 for more details.

### Analyses of cases and controls without comorbidities - adjusted analyses

In this sample, the burden of somatic symptoms did not differ between cases (*n* = 143) and controls (*n* = 1322) by number of antibiotic therapies. Differences in mental health and depressive symptoms between cases and controls did not vary according to serological results. Furthermore, when analysing cases (*n* = 85) and controls (*n* = 291) with known previous LB, but without comorbidities, somatic symptoms did not differ by the number of antibiotic treatments. These observations were confirmed by applying the multiple-imputation model. More details are shown in Tables S7-S10 and Figures S5-S8.

### Missing data

Little’s test for MCAR was not significant for cases, suggesting MCAR. For controls, the test was significant (*p* = 0.002), indicating potential non-MCAR, despite the small proportion of missing data (< 4%). The complete case and imputed analyses showed good concordance, supporting the assumption that missing data are likely MCAR or have minimal impact on the analyses. See Table S11 for an overview of variables with missing data.

## Discussion

The main finding in this cross-sectional controlled study is that 0.15% of Norwegians reported persistent self-reported health complaints attributed to ticks or tick-borne diseases. The recruitment of participants through GPs resulted in very few responders. However, by recruiting through SMS, we received 392 of the 500 responses. The health complaints attributed to tick-borne diseases were substantial, but the exposure registered to ticks and treatment for tick-borne diseases did not statistically affect the level of symptoms.

### Epidemiology

The prevalence of persistent posttreatment symptoms after LB range between 0% and 48% in other studies [36, 37]. In a Norwegian study, the post-infectious symptom load after EM was similar to that of the general population [38]. Although underreporting may appear, the incidence rate of laboratory verified LB in Norway in 2017 was 9 per 100,000 according to MSIS (EM not registered) and population data from NPRN. The prevalence numbers are clearly related to the estimate method and thus not easy to compare. However, the lower annual incidence rate of residual symptoms after LB suggests that these health problems are long-lasting and that overlapping conditions are difficult to rule out. For example, the cases had more comorbidities than the controls. Rheumatologic disease (24.0%) and chronic fatigue syndrome (16.5%) were the most prevalent. Systemic autoimmune joint disease after LB has been reported [39], and some of our participants may be affected. Furthermore, the population prevalence of similar conditions that often resemble the same symptoms as those in our cohort, such as chronic fatigue syndrome [40] (0.2-0.4%) and neuropsychiatric symptoms in long-COVID-19 [41] (0.1%), corresponds to our study.

### Recruitment approach

The three-way approach to the recruitment of study participants showed different results. The highest response rate and the best physical health outcomes were observed in participants recruited through SMS, while the far lowest response rate was observed in those recruited through GP. It is not clear whether this low response rate reflects a low occurrence of these health problems or just busy GPs that do not respond. Some participants may have felt unrecognized by healthcare professionals [14], leading to disappointment in the healthcare system and increased trust in random testimonies in media channels [42]. Complicated patient-doctor relationships [43] may contribute to higher SMS response rates, thus avoiding addressing this ‘controversial’ topic with primary physicians.

### Diagnostic uncertainties

The diagnostic basis for a previous episode of LB is uncertain for some of the cases in our study cohort, and the background level of subjective health complaints in the Norwegian population is high [44]. A prevalence of 3% medically unexplained symptoms (MUPS) was found in a cross-sectional study in GP offices in the tick-endemic region of Vest-Agder, southern Norway [45]. In a previous study on patients referred for LB without objective evidence of infection, several other diagnoses could explain their persistent symptoms [46]. Cases had a higher burden of somatic and affective symptoms, more fatigue, and a lower HRQoL than controls. These associations are corroborated by other studies [36, 47, 48], although the study populations and designs, including the outcome measures, differ from our methods.

### Implications for clinical practice

Antibodies to tick-borne pathogens were significantly more prevalent among cases than among controls, except *A. phagocytophilum* and *C. burnetii* IgG. In the tick-endemic region of Søgne [22], Norway, *Bb* antibodies and other tick-borne microbes were not associated with somatic symptoms. Reduced physical health and fatigue among our cases were not influenced by previous exposure to tick-borne diseases or the number of antibiotic treatments for tick-borne diseases. However, we do not know whether multiple treatments were prescribed due to true reinfections or due to health complaints of an unknown aetiology attributed to tick-borne diseases. We also do not know the duration of the treatment or the type of treatment the participants received. Studies have not shown clear improvements for patients with symptoms attributed to LB after repeated or long-term antibiotic treatments [19, 49–51]. Although untreated cases had a slightly higher burden of symptoms, they did not report more exposure to tick bites or tick-borne diseases compared to treated cases. However, untreated cases did report more comorbidities. Among the untreated cases, there was no greater burden of symptoms with increased exposure to ticks and tick-borne diseases. However, 43% of untreated cases reported EM. A study reported that approximately 50% of EM patients are seronegative [2], and another showed that 23% of EM patients were overlooked by physicians [52]. In the Søgne cohort [21], the association between somatic symptoms and exposure to tick bites and EM was weak. Immunocompetent persons will often resolve a borrelia infection independently of antibiotic treatment. However, we cannot exclude the possibility that some of our cases have undergone untreated LB with subsequent persistent symptoms. A prolonged untreated tick-borne infection lasting more than 6 months should result in increased *Bb*-IgG seropositivity if the person is immunocompetent. Furthermore, by removing comorbidities, the number of antibiotic treatments, tick bites, and serological results did not change the differences between cases and controls in any outcome. This supports the idea that comorbidities with baseline lower function and psychosocial mechanisms can influence the path from infection to persistent symptoms more than tick bites or tick-borne diseases itself, as outlined in previous studies [47]. In adjusted analyses, *Bb* seropositive cases of *Bb* had reduced mental health and depressive symptoms, not observed in controls (Fig. 2). A similar finding was observed in a Czech study [53]. In a large Danish cohort study [54], the synergistic effect of inflammatory processes and infections, autoimmunity, and psychological factors increased the risk of mental problems after infections, with infection alone being the most prominent risk factor. A population-based Danish cohort study [48] on associations between LB and mental health found that mental disorder rates were higher after LB compared to those without a history of LB. The rates of mental disorders increased with increasing number of LB episodes and with temporal proximity to diagnosis, but the absolute risk in the population was low. However, most of the study participants in this Danish study were diagnosed with LB using ICD-10 codes without further verification. Our data may align with the two Danish cohort studies, but we cannot determine whether the decline in mental health occurred after tick exposure. Another recent Danish study on verified LNB patients found no correlation between LNB and psychiatric symptoms [55]. Therefore, our findings might suggest that the cumulative effect of comorbidities and seropositivity to *Bb* could play a contributing role in the association between mental illness and persistent health complaints attributed to tick bites or tick-borne diseases. However, clinicians should carefully look for other causes of the symptoms when assessing such patients.

### Policy considerations and future research directions

Although the level of exposure is high among those who believe they have persistent symptoms attributed to tick bites, it is not certain that this is the underlying cause of the symptoms. These findings may be relevant for the development of new patient and treatment guidelines. Additionally, our results lay the groundwork for further research, for example, on the effects of physical activity and cognitive behavioral therapy. It would also be interesting to investigate the impact of various comorbidities in more detail.

### Strengths and limitations

The strength of our study lies in its nationwide population-based design, that incorporates a large and diverse sample. Participants were recruited through three different methods, ensuring representation of most people in Norway who attribute their persistent symptoms to tick-borne diseases. The study also benefits from a high response rate, and a wide range of validated questionnaires assessing symptoms and health quality from various perspectives. Controls came from a tick-endemic region, answered the same questionnaires, and blood samples were analysed by the same laboratory. A multiple-imputation model was applied, but missing data had little impact on the analyses. The current study has limitations due to its retrospective design, which can introduce recall bias and misclassifications in survey responses. The controls were recruited from an endemic tick area and are not representative of the general population. However, Norway’s demographics are largely uniform, with minor variations in age and immigration, which were accounted for in the analysis. Although recruitment was carried out at different times, minimal changes in demographics and tick exposure were observed. The control group had normal somatic symptoms, physical health and fatigue, but slightly more anxiety and a lower mental health score than the general population. This may limit the generalizability of mental health assessment, making it more relevant for people with existing mild psychological difficulties. However, the lack of associations between tick exposure and health outcomes is a finding that should be relevant to broader populations, including low-endemic areas. Different recruitment methods for the cases may have introduced selection bias, creating a heterogeneous study population. For example, SMS recruits had better physical health than those invited through NLBA and the website. However, the population with persistent symptoms attributed to tick-borne diseases is also inherently heterogeneous. Therefore, the various recruitment methods have been advantageous by increasing case access and improving statistical power. Rigorous statistical adjustments for demographic and health-related factors help mitigate the impact of selection bias. Furthermore, we do not know whether all cases recruited by SMS and invitation have been evaluated by their GP for a history of LB. It is not clear whether all recipients opened their SMS, but it is unlikely that this was a frequent problem. However, the SMS recruitment method may have led to an underrepresentation of older, chronically ill individuals and persons from socioeconomically challenged groups.

## Conclusions

The crude prevalence of persistent health problems in Norway attributed to tick bites or tick-borne diseases is 0.15%. The cases reported significantly poorer physical health and increased fatigue compared to controls. These relationships were not affected by tick exposures and prior treatments. Cases that were never treated for tick-borne diseases did not have higher occurrences of self-reported tick-borne diseases and IgG antibodies compared to treated cases. However, poorer mental health in cases may be associated with *Bb* seropositivity, especially for the ones with comorbidities. In conclusion, no clear associations were found between tick bites, tick-borne diseases and persistent health complaints.

## Electronic supplementary material

Below is the link to the electronic supplementary material.

Supplementary Material 1

Supplementary Material 2

## Data Availability

The datasets generated and/or analysed during the current study are not publicly available due to privacy concerns related to General Data Protection Regulation (GDPR) and the potential for re-identification. However, they can be obtained from the corresponding author upon reasonable request.

## Abbreviations

ANOVA: Analysis of Variance
Bb: Borrelia burgdorferi sensu lato
EM: Erythema migrans
EMM: Estimated marginal means
FSS: The Fatigue Severity Scale
GDPR: General Data Protection Regulation
GP: General practitioner
HADS: The Hospital Anxiety and Depression Scale
HRQoL: Health-related quality of life
LB: Lyme borreliosis
LNB: Lyme neuroborreliosis
MCAR: Missing completely at random
MCS: Mental component summary
MHW: The Modern Health Worries Questionnaire
MSIS: The Norwegian Surveillance System for Communicable Diseases
MUPS: Medically unexplained symptoms
NIPH: National Institute of Public Health
NLBA: Norwegian Lyme Borreliosis Association
NPRN: Norwegian National Population Registry
OR: Odds ratio
PCS: Physical component summary
PHQ-15: The Patient Health Questionnaire-15
PROMs: Patient-reported outcome measures
PTLDS: Post Lyme disease syndrome
TBEV: Tick-borne encephalitis virus
