# Supplementary material for: Persistent self-reported health complaints in Norwegians who attribute their symptoms to tick bites or tick-borne disease– a cross-sectional controlled study
Source: BMC Infect Dis. 2025 May 16;25:711. doi: 10.1186/s12879-025-11104-0 (PMC12085041; doi:10.1186/s12879-025-11104-0)
Supplement: Supplementary file 1 — Supplementary Material 1 [file 12879_2025_11104_MOESM1_ESM.docx]

| **(Supplementary questionnaire)**  **BorrSci WP1**   **QUESTIONNAIRE IN CONNECTION WITH PARTICIPATION IN BorrSci**  **Short brief:**  Thank you for agreeing to participate in BorrSci.(Lyme borreliosis; a scientific approach to reducing diagnostic and treatment uncertainties): Work Package 1: Chronic Lyme Borreliosis: Epidemiology, Clinical Aspects, and Laboratory Findings. The survey will take approximately 15-30 minutes to complete. It is important that you try to answer as accurately and thoroughly as possible.  We, who receive your responses, will not interpret them in relation to disease symptoms. If you experience health concerns based on your answers, we recommend discussing them with your general practitioner.  **BACKGROUND**  BorrSci stands for Borrelia Science: it is a large research project aimed at improving the diagnosis and treatment of borreliosis, as well as increasing knowledge about the phenomenon of chronic borreliosis. The research project is a collaboration between several health enterprises and research institutions in Norway. Work Package 1 addresses chronic Lyme borreliosis and will examine: epidemiology, clinical aspects, and laboratory findings. You can find more information about the project here: www.fhi.no/borrsci.  In this study, the Norwegian Institute of Public Health aims to gain an overview of how common chronic borreliosis is, map symptoms and signs of chronic borreliosis, and analyze blood samples to detect any tick-borne infections. The results of the study will be published in international scientific journals and on the websites of the Norwegian Institute of Public Health and the Tick Center (Norwegian National Advisory Unit on Tick-borne Diseases).  An important part of the study is to respond to a questionnaire regarding your health status, as well as some questions about socioeconomic conditions. The purpose of these questions is to gain an overview of your health status and factors that may influence it.  You have previously consented to participate in this study on chronic issues following tick bites, and you are therefore receiving this survey.  We ask that you answer all questions in the questionnaire. It will take between 15 and 30 minutes to complete the survey.  **PRIVACY**  All health information you provide us through the questionnaires and results from your blood sample analyses will be coded, meaning it will be processed without names, birth numbers, or other directly identifying information. Only authorized personnel associated with the project will have access to the code and will be able to trace it back to you. It will not be possible to identify you in the results of the study when they are published.  The project has been approved by the Regional Committee for Medical and Health Research Ethics and has been approved by the data protection officer regarding GDPR (General Data Protection Regulation).  Participation in the study is voluntary, and you can withdraw from the study at any time without providing a reason. You can also request the deletion of collected information and biological samples about you.  If you wish to withdraw from the study, you can contact X, who is the project leader. Send him an email at audun.aase@fhi.no or contact him by phone at XX XX XX XX  **GUIDANCE FOR THE QUESTIONNAIRE**  It is important that you try to answer as accurately and completely as possible. If you experience health concerns through your responses, we recommend discussing them with your general practitioner.  You can navigate back and forth in the questionnaire by clicking the arrow buttons at the bottom of the page. You can interrupt your response at any time and later return to the questionnaire to complete it. Once you have answered the entire questionnaire and reached the final page, your response will be marked as “completed” and cannot be changed, for your privacy.  If you wish to review the questions before answering, you have the option to print the questionnaire by clicking the printer icon below.  For questions regarding this questionnaire, please contact X (email: xxxx.xxxxx@xxx.xx, phone: xx xx xx xx).  Thank you for your participation, and good luck! |
| --- |

| **Background information** |
| --- |

Name
*** This applies only to those responding by ordinary mail

_____

**Age at last birthday (years)**

_____

Hight:


Please provide your response in centimeters. 

________________________________________

Vekt:


Please provide your response in kilograms. 

________________________________________

Sex

(1) ❑ Female

(2) ❑ Male

Nationality

(1) ❑ Norwegian

(2) ❑ **From the Nordic countries excluding Norway**

(3) ❑ From Europe excluding the Nordic countries

(4) ❑ From Asia

(5) ❑ From Africa

(6) ❑ From North-America

(7) ❑ From South-America

(8) ❑ From Oceania

Net total income in your household

(1) ❑ Less than 20.000 NOK per month

(2) ❑ 20.000-40.000 NOK per month

(3) ❑ More than 40.000 NOK per month

Do you live alone?

(1) ❑ Yes

(2) ❑ No

If no, who do you live with?

(1) ❑ Partner

(2) ❑ Children

(3) ❑ Parents

(4) ❑ Other family

(5) ❑ Friends

(6) ❑ Others

(7) ❑ None of the items

| **Work and education** |
| --- |

Education after primary school:

(1) ❑ Student

(2) ❑ Three years or less

(3) ❑ Between three and six years

(4) ❑ More than six years

Current employment status (multiple choices are possible simultaneously):

(1) ❑ Fully employed

(2) ❑ Partially employed

(3) ❑ Pensioner

(4) ❑ Student

(5) ❑ Stay-at-home

(6) ❑ Job seeker

(7) ❑ Fully sick-listed

(8) ❑ Partially sick-listed

(9) ❑ Fully disabled pensioner

(10) ❑ Partially disabled pensioner

(11) ❑ None of the items

Have you been fully or partially sick-listed for more than a month continuously in the last two years?

(1) ❑ Yes

(2) ❑ No

| **Activity level** |
| --- |

**Average number of hours of physical activity per week (running, hiking, strength**

**training, cycling, swimming, and similar activities).**

(1) ❑ Less than 1

(2) ❑ 1 or more, but less than 3

(3) ❑ 3 or more, but less than 6

(4) ❑ 6 or more

| **Previous tick bites and tick-borne diseases** |
| --- |

Have you ever had a tick bite?

(1) ❑ Yes, once

(2) ❑ Yes, twice

(3) ❑ Yes, more than twice

(4) ❑ No

Have you had a tick bite in the last year?

(1) ❑ Yes

(2) ❑ No

**Have you ever had a red ring-shaped rash (larger than about 5 cm) around a tick bite**

**(erythema migrans)?**

(1) ❑ Yes, once

(2) ❑ Yes, twice

(3) ❑ Yes, more than twice

(4) ❑ No

**Have you ever had any other tick-borne disease besides erythema migrans?**

**(multiple choices are possible simultaneously)**

(1) ❑ Yes, neuroborreliosis

(2) ❑ Yes, Lyme arthritis

(3) ❑ Yes, another form of Borrelia disease

(4) ❑ Yes, tick-borne encephalitis (TBE)

(5) ❑ Yes, another tick-borne disease.

(6) ❑ No

**Do you have health issues that you or your doctor believe are caused by an infection**

**with Borrelia or other tick-borne microbes?**

(1) ❑ Yes

(2) ❑ No

| **Previous laboratory tests regarding tick-borne diseases** |
| --- |

**Have you ever had a blood test to check for tick-borne diseases?**

(1) ❑ Yes, but I never received the answer.

(2) ❑ Yes, but I don’t remember the answer I received.

(3) ❑ Yes, but I didn’t understand the answer I received.

(4) ❑ Yes, the tests were normal/negative.

(5) ❑ Yes, antibodies against Borrelia were detected.

(6) ❑ Yes, antibodies against tick-borne encephalitis (TBE) were detected.

(7) ❑ Yes, antibodies against both Borrelia and TBE were detected.

(8) ❑ Yes, antibodies against other tick-borne diseases were detected.

(9) ❑ I don’t know.

(10) ❑ No

**Have you ever had a spinal fluid test (‘lumbar puncture’) to check for tick-borne**

**diseases?**

(1) ❑ Yes, but I don’t know the answer.

(2) ❑ Yes, the test was normal/negative.

(3) ❑ Yes, the test showed that I had neuroborreliosis.

(4) ❑ Yes, the test showed another disease.

(5) ❑ I don’t know.

(6) ❑ No

**How satisfied are you with how the public healthcare system handled your condition?**

(3) ❑ Very satisfied

(2) ❑ Somewhat satisfied

(1) ❑ Not satisfied

**Have you been diagnosed with Lyme disease or another tick-borne illness at**

**laboratories outside the public healthcare system? (You can answer for both Norway**

**and abroad).**

(1) ❑ Yes, in Norway

(2) ❑ Yes, abroad

(3) ❑ Yes, both in Norway and abroad

(4) ❑ No

Please answer the following:

| Which country? | _____ |
| --- | --- |
| Which microbe/agent was found? | _____ |

**How satisfied are you with how the alternative healthcare service handled your**

**condition?**

(3) ❑ Very satisfied

(2) ❑ Somewhat satisfied

(1) ❑ Not satisfied

**Reviewed vaccination and treatment for tick-borne diseases.**

**Have you ever been treated with antibiotics? (It is possible to check both of the last two answer options at the same time.)**

(1) ❑ No

(2) ❑ Yes, the last year

(3) ❑ Yes, previously

**Have you ever been treated with antibiotics for a tick-borne disease?**

(1) ❑ No, never

(2) ❑ Yes, one course

(3) ❑ Yes, two courses

(4) ❑ Yes, three courses

(5) ❑ Yes, four courses

(6) ❑ Yes, five or more courses

(7) ❑ I don’t remember

**Have you received any other treatment for your Lyme disease besides through the public**

**healthcare system? (Note: you can answer for both Norway and abroad if applicable.)**

(1) ❑ Yes, in Norway

(2) ❑ Yes, abroad

(3) ❑ Yes, both in Norway and abroad

(4) ❑ No

**Have you received long-term antibiotic treatment for your Lyme disease (> 4 weeks)?**

**(Note: you can answer for both Norway and abroad if applicable.)**

(1) ❑ Yes, in Norway

(2) ❑ Yes, abroad

(3) ❑ Yes, both in Norway and abroad

(4) ❑ No

****Are you vaccinated against tick-borne encephalitis (TBE)?****

(1) ❑ No

(2) ❑ Yes, one shot

(3) ❑ Yes, two shots

(4) ❑ Yes, fully vaccinated

Are you vaccinated against yellow fever?

(1) ❑ Yes

(2) ❑ No

(3) ❑ I don’t know

| **Other diseases** |
| --- |

**Have you been diagnosed with any other diseases? (Multiple choices are possible at**

**the same time.)**

(1) ❑ No

(2) ❑ Parkinsons disease

(3) ❑ Multiple sclerosis

(4) ❑ Dementia

(5) ❑ Epilepsia

(6) ❑ Stroke

(7) ❑ Polyneuropathy

(8) ❑ Other disease(s) in the nervous system _____

(9) ❑ Arthritis

(10) ❑ Fibromyalgia

(11) ❑ Other diseases in joints/muscles

(12) ❑ Endocrine/metabolic disease

(13) ❑ Diabetes

(14) ❑ Depression/anxiety

(15) ❑ Other mental illness

(16) ❑ ME/Chronic fatigue syndrome

(17) ❑ Heart disease

(18) ❑ COPD/asthma

(19) ❑ Allergy

(20) ❑ Cancer

(21) ❑ Skin disease

(22) ❑ Eye disease

(23) ❑ Other diseases

**Do you take medication regularly? (Multiple choices are possible at the same time.)**

(1) ❑ No

(2) ❑ Medication for nervous system disease

(3) ❑ Medication for joint/muscle diseases

(4) ❑ Medication for mental illness

(5) ❑ Medication for heart and blood vessel conditions (blood pressure lowering, blood thinners, heart failure, etc)

(6) ❑ Medication for high cholesterol

(7) ❑ Medication for COPD or asthma

(8) ❑ Medication for endocrine or metabolic diseases

(9) ❑ Insulin

(10) ❑ Tablets for diabetes

(11) ❑ Other hormone medication (birth control pills, menopause medication, other hormonal diseases)

(12) ❑ Medication for allergies

(13) ❑ Medication for pain

(14) ❑ Medication for cancer (chemotherapy or other)

(15) ❑ Medication for other conditions

| **TOBACCO** |
| --- |

Do you use any form of tobacco?

Please check one or more of the options below

(1) ❑ Cigarettes: Number of cigarettes per day: ________________________________________

(2) ❑ Snus tobacco

(3) ❑ Chewing tobacco

(4) ❑ No
