# Supplementary material for: Persistent self-reported health complaints in Norwegians who attribute their symptoms to tick bites or tick-borne disease– a cross-sectional controlled study
Source: BMC Infect Dis. 2025 May 16;25:711. doi: 10.1186/s12879-025-11104-0 (PMC12085041; doi:10.1186/s12879-025-11104-0)
Supplement: Supplementary file 2 — Supplementary Material 2 [file 12879_2025_11104_MOESM2_ESM.docx]

***Supplementary material:***

Manuscript Title: «Persistent self-reported health complaints in Norwegians who attribute their symptoms to tick bites or tick-borne disease – a cross-sectional controlled study»

by Dahlberg et al.

| ***Table S1*: Comparison to pooled normative data for controls:** | | | | |
| --- | --- | --- | --- | --- |
|  | PHQ-15 | | PCS | |
|  | Controls | Normal | Controls | Normal |
| Number (n) | 2720 | 3406 | 2686 | 4861 |
| Mean | 5.03 | 6.3 | 48.84 | 42.3 |
| Standard deviaton | 4.358 | 4.6 | 9.55 | 9.3 |
|  | MCS * | | FSS | |
|  | Controls | Normal | Controls | Normal |
| Number (n) | 2686 | 4859 | 2720 | 1859 |
| Mean | 51.1 | 80.55 | 3.07 | 4.0 |
| Standard deviaton | 9.53 | 15.36 | 1.60 | 1.31 |
|  | HAD-D | | HAD-A * | |
|  | Controls | Normal | Controls | Normal |
| Number (n) | 2720 | 39573 | 2720 | 39277 |
| Mean | 2.9 | 3.325 | 4.42 | 4.016 |
| Standard deviaton | 3.04 | 2.887 | 3.505 | 3.287 |

* All outcome variables for the controls were significantly different from

normative data (*p*<0.001), with MCS (*) lower and HAD anxiety (*) higher than

normative data. There was no higher HAD-A score among controls compared to

normative data in a selected sample without comorbidities.

The same age groups were used for comparisons between controls and normative data,

but different groups were used for each outcome variable due to varying age groups in

the normative data: PHQ-15 (age 18-79 years), PCS and MCS (age 20-79 years),

FSS (age 18-80 years). The age range for the HAD score is not defined in the normative data.

***The multiple imputation model:***

**Imputed variables:**

Income ( < 20.000 NOK per month (net) = 0, > 20.000 NOK per month (net) = 1), Living alone (Alone = 1, Not alone = 2), Education after primary school (Student = 1, < 3 years = 2, 3-6 years = 3, over 6 years = 4), Sick leave more than two months last year (Yes = 1, No = 2), TBE vaccinated (No = 1, yes, one injection = 2, yes, two injections = 3, fully vaccinated = 4), Score PHQ-15, Score FSS, PCS, MCS, Score MHW, Score HAD anxiety, Score HAD depression, Physical activity (0 = < 3 hours per week, > 3 hours per week = 1), Tick bites ever (Never = 1, Once = 2, Twice or more = 3), Erythema migrans ( Never = 1, Once = 2, Twice or more = 3), Antibiotic treatments against a tick-borne disease ( Never = 0, Once = 1, Twice or more = 2), Tick-borne pathogens from serological analyses (No pathogens proven = 0, Bb alone = 1, More than one co-pathogen without Bb = 2, Bb and more than one co-pathogen = 3), Borrelia IgG (Negative = 0, Positive = 1), GROUP (Cases = 1, controls = 0), Gender (Male = 1, female = 0), Work (1 = Fully employed, Not fully employed = 0), One or more disseminated borrelia infections (Yes = 1, No = 0), Comorbidities (No diseases = 0, One disease = 1, Two or more diseases = 2), Age categories (18-39 = 1, 40-59 = 2, 60+ = 3).

**Variables with no missing data (in both cases and controls):**

**Cases:** GROUP (Cases = 1, controls = 0).

**Controls:** GROUP (Cases = 1, controls = 0), Gender (Male = 1, female = 0), Work (1 = Fully employed, Not fully employed = 0), One or more disseminated borrelia infections (Yes = 1, No = 0), Comorbidities (No diseases = 0, One disease = 1, Two or more diseases = 2), Age categories (18-39 = 1, 40-59 = 2, 60+ = 3).

We tried to impute the interaction terms: GROUP x Tick-borne pathogens from serological analyses, GROUP x Antibiotic treatments against a tick-borne disease, GROUP x Erythema migrans, GROUP x One or more disseminated borrelia infections, GROUP x Tick bites ever but the model did not converge, and the interaction terms were rejected in the model. Thus, instead of directly incorporating the interaction terms into the imputation model, we performed separate imputations for cases and controls, and then merged the two datasets.

We also tried to incorporate more variables in the model, but due to correlation between other variables these were rejected. Thus, the model utilized was considered eligible.

| ***Table S2***  **Multiple linear regressions** ^1^ **with interactions between the case-control dichotomy and antibiotic therapy on PHQ-15 with estimated marginal means** ^2^ **(EMM) and**  **95% confidence intervals (CI)** | | |  |  |  |
| --- | --- | --- | --- | --- | --- |
|  | **Cases** | **Controls** | **Interaction** ^2^ |  |  |
|  | EMM with 95% CI | EMM with 95% CI | *p*-value |  |  |
|  | *p*-value | *p*-value |  |  |  |
|  |  |  |  |  |  |
| ***Antibiotic therapy*** |  |  | NA |  |  |
| *Never (ref* ^3^*)* | 12.9 (11.7 – 14.1) | 5.5 (5.3 – 5.8) |  |  |  |
| *Once* | **10.7 (9.8 – 11.6)** | 5.6 (5.1 – 6.1) |  |  |  |
| *Twice or more* | 11.2 (10.4 – 12.1) | **6.5 (5.7 – 7.3)** |  |  |  |
|  |  |  | NA |  |  |
| ***Tick-bites***  *Never (ref* ^3^*)* | 13.5 (9.7 – 17.3) | 5.1 (4.6 – 5.5) |  |  |  |
| *Once* | **10.9 (9.7 – 12.1)** | **6.0 (5.5 – 6.5)** |  |  |  |
| *More than twice* | **11.3 (10.7 – 12.0)** | **5.6 (5.3 – 5.9)** |  |  |  |

^1^ Adjusted for age, gender, education, comorbidities, and physical activity.

^2^ The *p-*value for interaction was not calculated after multiple imputation for analyses of variance (ANOVA). See *p*-value from complete case analyses. NA means not-applicable.

^3^ The significant difference from the complete case analyses was also applied in the multiple imputation model and is indicated in bold text with reference categories as shown in the table.


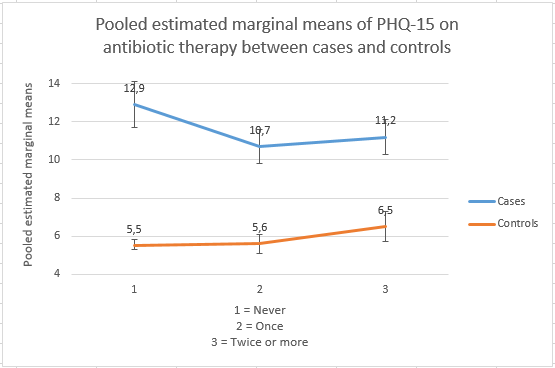


Figure S1. Multiple linear regression after multiple imputation with predictive mean matching of PHQ-15 on group x antibiotic therapy. Reference category is never.


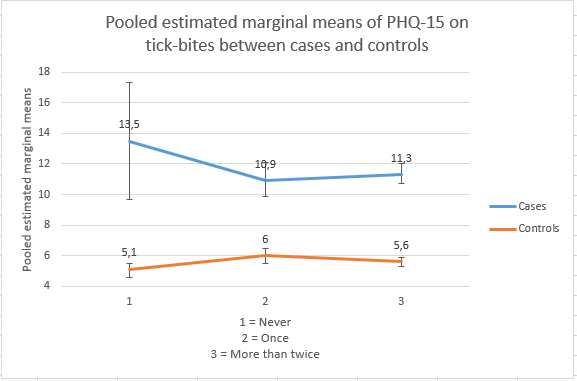


Figure S2. Multiple linear regression after multiple imputation with predictive mean matching of PHQ-15 on group x tick-bites. Reference category is never.

| ***Table S3***  **Multiple linear regressions** ^1^ **with interactions between the case-control dichotomy and antibiotic therapy on MCS with estimated marginal means** ^2^ **(EMM) and**  **95% confidence intervals (CI)** | | |  |  |  |
| --- | --- | --- | --- | --- | --- |
|  | **Cases** | **Controls** | **Interaction** ^2^ |  |  |
|  | EMM with 95% CI | EMM with 95% CI | *p*-value |  |  |
|  | *p*-value | *p*-value |  |  |  |
|  |  |  |  |  |  |
| ***Tick-borne pathogens*** |  |  | NA |  |  |
| *Negative (ref* ^3^*)* | 45.2 (43.5 – 47.0) | 49.6 (49.0 – 50.2) |  |  |  |
| *Bb* | **42.5 (40.5 – 44.5)** | **50.4 (49.5 – 51.3)** |  |  |  |
| *Co-pathogens*  *Bb and co-pathogens* | 45.4 (42.9 – 47.9)  **42.6 (40.2 – 45.0)** | 50.3 (48.7 – 51.8)  51.2 (49.0 – 53.6) |  |  |  |

^1^ Adjusted for age, gender, education, comorbidities, and physical activity.

^2^ The *p-*value for interaction was not calculated after multiple imputation for analyses of variance (ANOVA). See *p*-value from complete case analyses. NA means non-applicable.

^3^ The significant difference from the complete case analyses was also applied in the multiple imputation model and is indicated in bold text with reference categories as shown in the table.


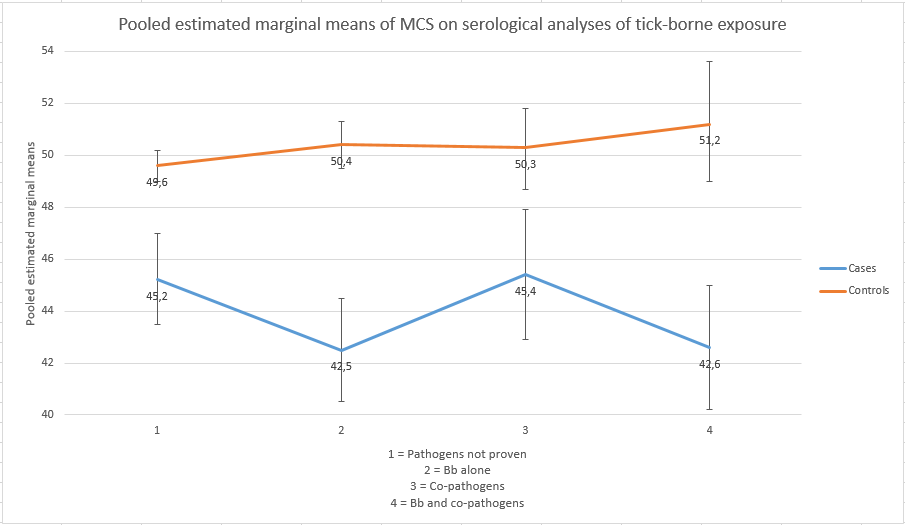


Figure S3. Multiple linear regression after multiple imputation with predictive mean matching of MCS on group x tick-borne infections. Reference category is pathogens not proven.

| ***Table S4***  **Multiple linear regressions** ^1^ **with interactions between the case-control dichotomy and antibiotic therapy on HAD depression with estimated marginal means** ^2^ **(EMM) and**  **95% confidence intervals (CI)** | | |  |  |  |
| --- | --- | --- | --- | --- | --- |
|  | **Cases** | **Controls** | **Interaction** ^2^ |  |  |
|  | EMM with 95% CI | EMM with 95% CI | *p*-value |  |  |
|  | *p*-value | *p*-value |  |  |  |
|  |  |  |  |  |  |
| ***Tick-borne pathogens*** |  |  | NA |  |  |
| *Negative (ref* ^3^*)* | 5.3 (4.7 – 5.9) | 3.3 (3.1 – 3.5) |  |  |  |
| *Bb* | **6.0 (5.3 – 6.7)** | 3.2 (2.9 – 3.5) |  |  |  |
| *Co-pathogens*  *Bb and co-pathogens* | 4.7 (3.7 – 5.6)  5.2 (4.3 – 6.0) | 3.5 (3.0 – 4.0)  3.2 (2.4 – 4.0) |  |  |  |

^1^ Adjusted for age, gender, education, comorbidities and physical activity.

^2^ The *p-*value for interaction was not calculated after multiple imputation for analyses of variance (ANOVA). See *p*-value from complete case analyses. NA means non-applicable.

^3^ The significant difference from the complete case analyses was also applied in the multiple imputation model and is indicated in bold text with reference categories as shown in the table.


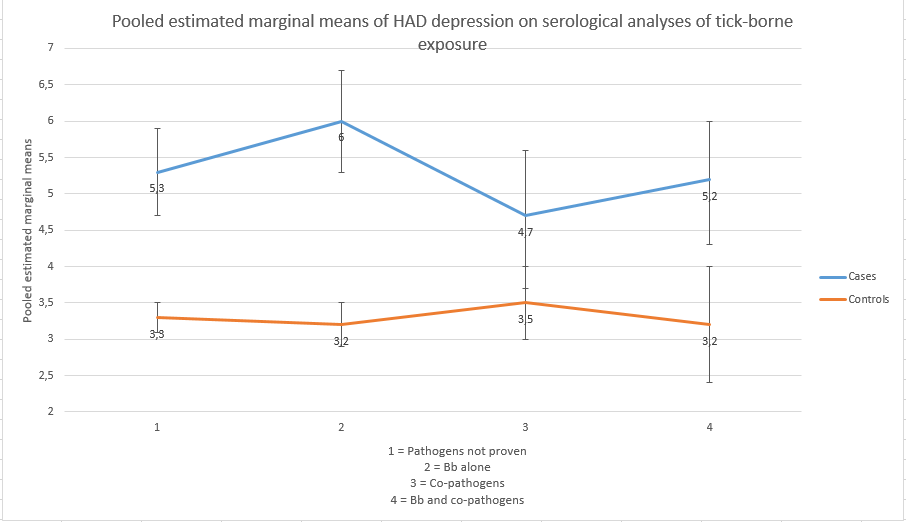


Figure S4. Multiple linear regression after multiple imputation with predictive mean matching of HADS depression on group x tick-borne infections. Reference category is pathogens not proven.

| ***Table S5***: Cases | Untreated (N= 81) | Treated (N= 322) | P value |
| --- | --- | --- | --- |
| One or more tick-bites | 78/81 (96.3%) | 314/321 (97.8%) | P=0.432 |
| Bb-IgG alone | 18/69 (26.1%) | 75/274 (27.4%) | P=0.830 |
| Other tick-borne serology alone | 9/69 (13.0%) | 43/274 (15.7%) | P=0.583 |
| Bb-IgG and other tick-borne serology combined | 5/69 (7.2%) | 59/274 (21.5%) | **P = 0.006** |
| One or more episodes with EM | 34/79 (43%) | 209/317 (65.9%) | ***p* < 0.001** |
| LNB by lumbar puncture | 1/81 (1.2%) | 50/320 (15.6%) | ***p* < 0.001** |
| Lyme arthritis | 4/77 (5.2%) | 44/297 (14.8%) | ***p* = 0.025** |
| One or more comorbid disease | 63/76 (82.9%) | 208/292 (71.2%) | **P = 0.04** |
| Age (mean 95% CI) | 51.0 [47.6 – 54.5] | 55.1 [53.5 – 56.6] | **P=0.029** |
| HAD-depression>= 8 | 25/80 (31.3%) | 81/321 (25.2%) | *p*=0.275 |
| HAD-anxiety >= 8 | 29/80 (36.3%) | 92/321 (28.7%) | *P=0.186* |
| FSS >= 4 | 62/73 (84.9%) | 236/291 (81.1%) | *P=0.447* |
| PHQ >= 10 | 52/72 (72.2%) | 163/294 (55.4%) | ***P= 0.01*** |
| PCS<50 | 75/81 (92.6%) | 290/322 (90.61%) | *P=0.396* |
| MCS<50 | 51/81 (63.0%) | 220/322 (68.3%) | *P=0.358* |
| MHW (mean 95% CI) | 1.98 [1.82 – 2.14] | 2.00 [1.91 – 2.10] | *P=0.824* |

Table for section 3.6. «Treated» implies receiving one or more antibiotic treatments for a tick-borne disease. Statistical test such as Chi-square test, Fishers exact test, or Students T-test were used as appropriate.

| ***Table S6***: | Yes | No | p-value |
| --- | --- | --- | --- |
| One or more tick-bites | 14.0 [9.0 – 18.0] | 23.0 [20.0 – 25.0] | **0.015** |
| Bb-IgG seropositive | 11.0 [7.0 – 14.0] | 15.0 [10.0 – 18.0] | **0.029** |
| Other antibodies to tick-borne disease | 13.0 [5.0 – 18.0] | 14 [9.0 – 18.0] | 0.737 |
| Seropositive Bb-IgG and other antibodies to tick-borne disease | 15.5 [8.0 – 17.5] | 13.5 [8.0 – 18.0] | 0.967 |
| Disseminated borr | 15.0 [10.0 – 20.0] | 14.0 [8.5 – 17.0] | 0.359 |
| EM | 15.0 [10.5 – 19.0] | 13.0 [7.0 – 17.0] | 0.247 |
| One or more comorbid disease | 14.5 [9.5 – 18.5] | 11 [7.0 – 18.0] | 0.229 |

Table for section 3.6. PHQ-15 among untreated cases. Medians with interquartile range (IQR) were calculated using Tukey’s Hinges method, and comparisons were made using the Mann-Whitney U test.

***Selected analyses:***

The outcome measures between cases and controls did not differ based on the presence or absence of a history of borreliosis. We aimed to investigate the interactions between group and tick-borne exposures in relation to MCS and HAD depression, as well as the interaction between group and antibiotic therapy in relation to PHQ-15, within selected groups considering comorbidities and self-reported borreliosis status. All outcome measures were stratified by comorbidities and borreliosis status, adjusted for age, gender, education, and physical activity:

***Known previous borreliosis and no comorbidities:***

MCS on tick-borne exposure: No interaction

HAD depression on tick-borne exposure: P interaction = 0.017 **(Table S7 and Fig S5)**

PHQ-15 on antibiotic therapy: No interaction

| ***Table S7***  **Multiple linear regressions** ^1^ **with interactions between the case-control dichotomy and tick-borne exposures on HAD depression with estimated marginal means** ^2^ **(EMM) and 95% confidence intervals (CI)** | | |  |  |  |
| --- | --- | --- | --- | --- | --- |
|  | **Cases** | **Controls** | **Interaction** ^1^ |  |  |
|  | EMM with 95% CI | EMM with 95% CI | Adjusted R^2^ |  |  |
|  | *p*-value | *p*-value | *p*-value |  |  |
|  |  |  |  |  |  |
| ***Tick-borne***  ***pathogens*** |  |  | 0.138  **0.017** |  |  |
| *Negative (ref* ^3^*)* | 6.0 (4.7 – 7.3) | 3.3 (3.1 – 3.5) |  |  |  |
| *Bb* | 4.8 (3.5 – 6.1) | 3.2 (2.9 – 3.5) |  |  |  |
| *Co-pathogens* | **2.4 (0.5 – 4.4)**  **0.003** | 3.5 (3.0 – 4.0) |  |  |  |
| *Bb and co- pathogens* | 5.7 (4.0 – 7.4) | 3.3 (2.5 – 4.1) |  |  |  |

^1^ The *p-*value for interaction is calculated for the F-statistic in analyses of variance (ANOVA) for the total model adjusted for age, gender, education, and physical activity. This model is selected on no comorbid diseases and known previous borreliosis.

^2^ Estimated marginal means (EMM) for the interaction term with 95 % confidence intervals.

^3^ Reference category (ref)for unadjusted pairwise comparisons (least significant difference) and corresponding *p*-value reported if significant (alfa <0.05). If significant corresponding *p*-value and EMM are reported in bold text as appropriate.


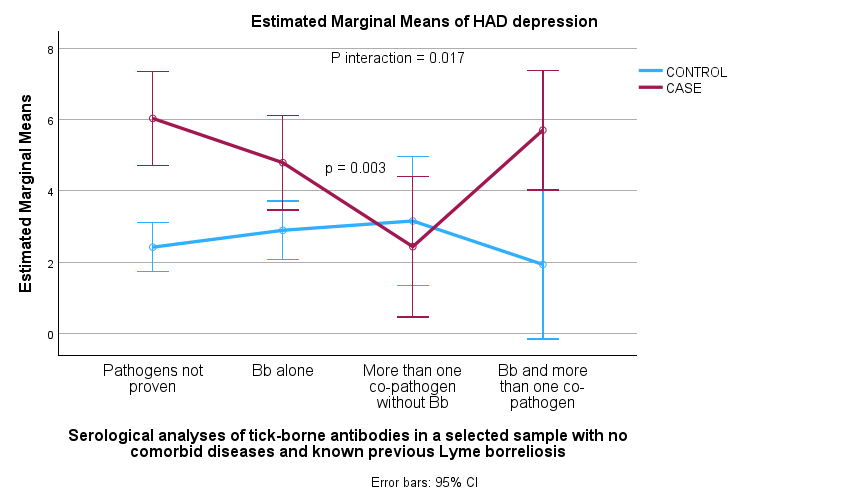


Figure S5: Multiple linear regression: Selected sample of no comorbid diseases and known previous Lyme borreliosis.

***Selected analyses excluding comorbidities:***

MCS on tick-borne exposure: No interaction

HAD depression on tick-borne pathogens: P interaction = 0.008 **(Table S8 and Fig S6)**

PHQ-15 on antibiotic therapy: No interaction

| ***Table S8***  **Multiple linear regressions** ^1^ **with interactions between the case-control dichotomy and tick-borne exposures on HAD depression with estimated marginal means** ^2^ **(EMM) and 95% confidence intervals (CI)** | | |  |  |  |
| --- | --- | --- | --- | --- | --- |
|  | **Cases** | **Controls** | **Interaction** ^1^ |  |  |
|  | EMM with 95% CI | EMM with 95% CI | Adjusted R^2^ |  |  |
|  | *p*-value | *p*-value | *p*-value |  |  |
|  |  |  |  |  |  |
| ***Tick-borne***  ***pathogens*** |  |  | 0.066  **0.008** |  |  |
| *Negative (ref* ^3^*)* | 6.5 (4.3 – 6.4) | 2.4 (2.2 – 2.7) |  |  |  |
| *Bb* | 5.1 (4.0 – 6.1) | 2.5 (2.1 – 2.8) |  |  |  |
| *Co-pathogens* | **2.5 (1.1 – 4.0)**  **0.002** | 2.7 (2.0 – 3.5) |  |  |  |
| *Bb and co- pathogens* | 5.5 (4.1 – 6.8) | 2.4 (1.4 – 3.4) |  |  |  |

^1^ The *p-*value for interaction is calculated for the F-statistic in analyses of variance (ANOVA) for the total model adjusted for age, gender, education and physical activity. This model is selected on no comorbid diseases.

^2^ Estimated marginal means (EMM) for the interaction term with 95 % confidence intervals.

^3^ Reference category (ref)for unadjusted pairwise comparisons (least significant difference) and corresponding *p*-value reported if significant (alfa <0.05). If significant corresponding *p*-value and EMM are reported in bold text as appropriate.


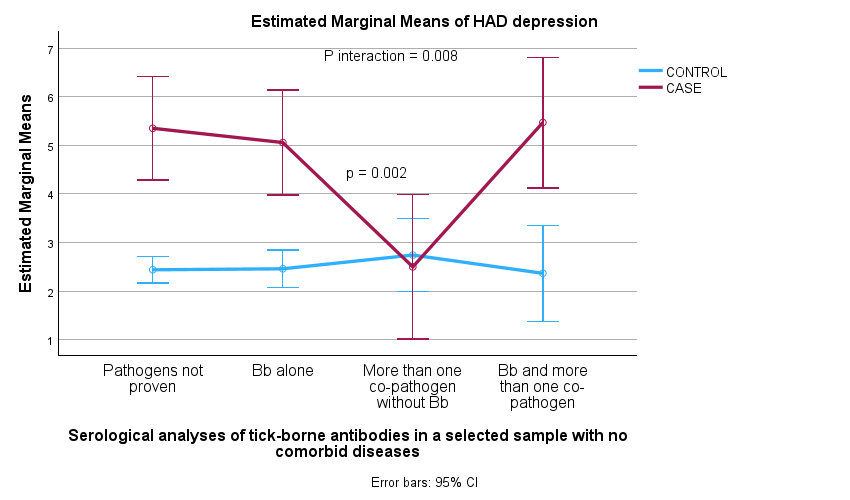


Figure S6: Multiple linear regression: Selceted sample of no comorbid diseases.

***Complete case analyses compared to the multiple imputation model:***

In the complete case analyses of persons with a history of Lyme borreliosis and no comorbidities, a significant interaction was found for HAD depression. Consequently, the same analysis was performed using the multiple imputation model, as shown below:

***Known previous borreliosis and no comorbid disease:***

**Complete case analyses:**

MCS on tick-borne exposure: No interaction

HAD depression on tick-borne exposure: P interaction = 0.017 **(Table S7 and Fig 5)**

PHQ-15 on antibiotic therapy: No interaction

**Multiple imputation model:**

| ***Table S9***  **Multiple linear regressions** ^1^ **with interactions between the case-control dichotomy and tick-borne pathogens on HAD depression with estimated marginal means** ^2^ **(EMM) and**  **95% confidence intervals (CI)** | | |  |  |  |
| --- | --- | --- | --- | --- | --- |
|  | **Cases** | **Controls** | **Interaction** ^2^ |  |  |
|  | EMM with 95% CI | EMM with 95% CI | *p*-value |  |  |
|  | *p*-value | *p*-value |  |  |  |
|  |  |  |  |  |  |
| ***Tick-borne pathogens*** |  |  | NA |  |  |
| *Negative (ref* ^3^*)* | 6.1 (4.6 – 7.5) | 2.4 (1.8 – 3.1) |  |  |  |
| *Bb* | 5.6 (4.2 – 6.9) | 3.1 (2.2 – 3.9) |  |  |  |
| *Co-pathogens*  *Bb and co-pathogens* | **3.9 (1.8 – 6.0)**  5.6 (3.9 – 7.4) | 3.1 (1.3 – 4.9)  2.1 (0 – 4.3) |  |  |  |

^1^ Adjusted for age, gender, education, and physical activity.

^2^ The *p-*value for interaction was not calculated after multiple imputation for analyses of variance (ANOVA). See *p*-value from complete case analyses. NA means non-applicable.

^3^ The significant difference from the complete case analyses is reported in bold text with reference category as shown in the table.

***No comorbidities:***

**Complete case analyses:**

MCS on tick-borne exposure: No interaction

HAD depression on tick-borne exposure: P interaction = 0.008 **(Table S8 and Fig 6)**

PHQ-15 on antibiotic therapy: No interaction

**Multiple imputation model:**

| ***Table S10***  **Multiple linear regressions** ^1^ **with interactions between the case-control dichotomy and tick-borne pathogens on HAD depression with estimated marginal means** ^2^ **(EMM) and**  **95% confidence intervals (CI)** | | |  |  |  |
| --- | --- | --- | --- | --- | --- |
|  | **Cases** | **Controls** | **Interaction** ^2^ |  |  |
|  | EMM with 95% CI | EMM with 95% CI | *p*-value |  |  |
|  | *p*-value | *p*-value |  |  |  |
|  |  |  |  |  |  |
| ***Tick-borne pathogens*** |  |  | NA |  |  |
| *Negative (ref* ^3^*)* | 5.7 (4.5 – 6.9) | 2.5 (2.2 – 2.8) |  |  |  |
| *Bb* | 5.6 (4.5 – 6.8) | 2.5 (2.1 – 2.9) |  |  |  |
| *Co-pathogens*  *Bb and co-pathogens* | **3.8 (1.9 – 5.6)**  5.4 (3.8 – 7.0) | 2.7 (2.0 – 3.5)  3.2 (1.4 – 3.4) |  |  |  |

^1^ Adjusted for age, gender, education, and physical activity.

^2^ The *p-*value for interaction was not calculated after multiple imputation for analyses of variance (ANOVA). See *p*-value from complete case analyses. NA means non-applicable.

^3^ The significant difference from the complete case analyses was also applied in the multiple imputation model and is indicated in bold text with reference categories as shown in the table.


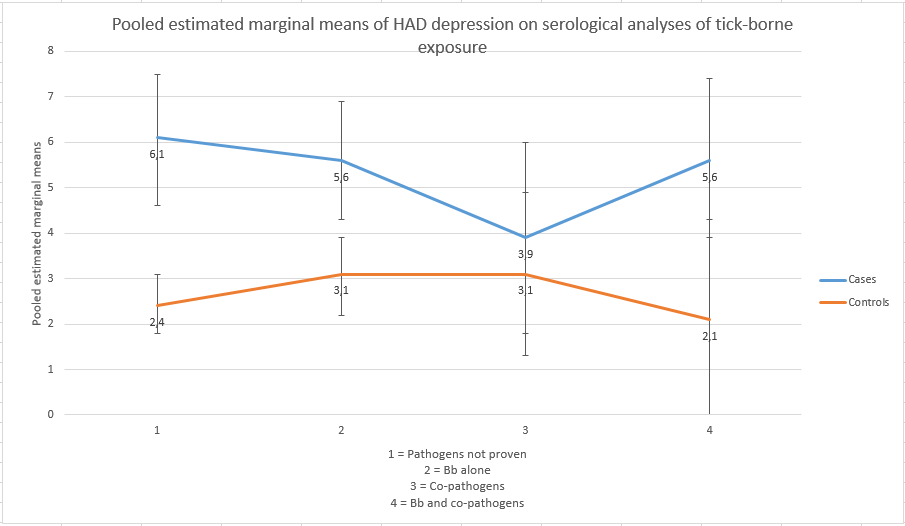


Figure S7. Multiple linear regression after multiple imputation with predictive mean matching of HADS depression on group x tick-borne infections in selected sample of no comorbid diseases and known Lyme borreliosis. Reference category is pathogens not proven.


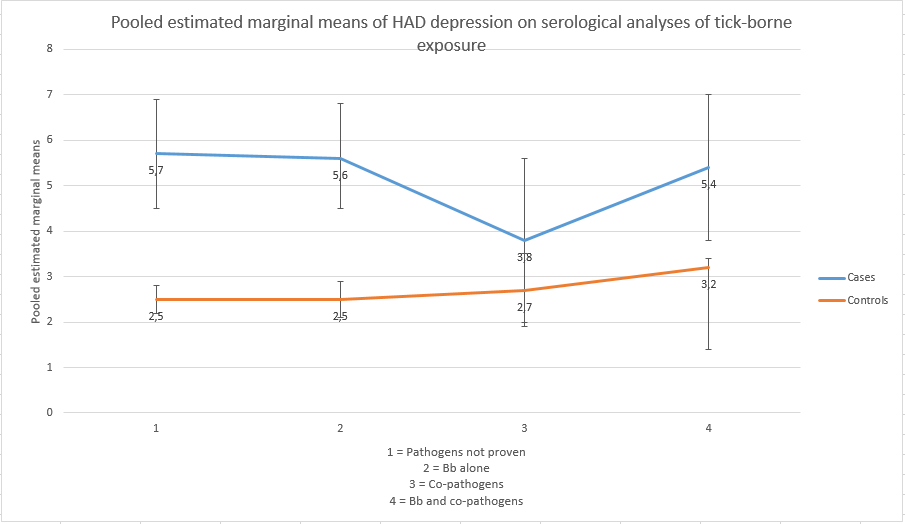


Figure S8. Multiple linear regression after multiple imputation with predictive mean matching of HADS depression on group x tick-borne infections in selected sample of no comorbid diseases. Reference category is pathogens not proven.

| ***Table S11***  **Variables with missing data: The number of missing values and the percentage of missing values per category are presented for cases (n=470) and controls (n=2803).** | | |  |  |
| --- | --- | --- | --- | --- |
|  | **Cases** | **Controls** |  |  |
|  | No. (%) | No. (%) |  |  |
|  |  |  |  |  |
| *PHQ-15* | 96 (20.4) | 7 (0.2) |  |  |
| *FSS* | 58 (12.3) | 44 (1.6) |  |  |
| *PCS* | 63 (13.4) | 41 (1.5) |  |  |
| *MCS* | 63 (13.4) | 41 (1.5) |  |  |
| *MHW*  *HADS anxiety*  *HADS depression*  *Income*  *Living alone*  *Education* | 58 (12.3)  58 (12.3)  58 (12.3)  53 (11.3)  50 (10.6)  54 (11.5) | 62 (2.2)  45 (1.6)  46 (1.6)  9 (0.3)  9 (0.3)  16 (0.6) |  |  |
| *Sick leave* | 57 (12.1) | 16 (0.6) |  |  |
| *TBE vaccination* | 81 (17.2) | 6 (0.2) |  |  |
| *Physical activity*  *Erythema migrans* | 50 (10.6)  57 (12.1) | 6 (0.2)  11 (0.4) |  |  |
| *Antibiotic therapy* | 67 (14.3) | 98 (3.5) |  |  |
| *Serology* | 85 (18.1) | 3 (0.1) |  |  |
| *Work-status* | 55 (11.7) | 0 (0) |  |  |
| *Comorbidities* | 50 (10.6) | 0 (0) |  |  |
| *Tick-bites* | 51 (10.9) | 6 (0.2) |  |  |
| *Disseminated borreliosis* | 32 (6.8) | 0 (0) |  |  |
| *Age* | 78 (16.6) | 0 (0) |  |  |

Little’s MCAR* test for both cases and controls combined was not significant (*p* = 0.063)

Little’s MCAR* test for cases was not significant (*p* = 0.223)

Little’s MCAR* test for controls was significant (*p* = 0.002)

*MCAR = missing at complete random.
